# Application of Gum Arabic and Methyl Cellulose Coatings Enriched with Thyme Oil to Maintain Quality and Extend Shelf Life of “Acco” Pomegranate Arils

**DOI:** 10.3390/plants9121690

**Published:** 2020-12-01

**Authors:** Tatenda Gift Kawhena, Alemayehu Ambaw Tsige, Umezuruike Linus Opara, Olaniyi Amos Fawole

**Affiliations:** 1 Department of Horticultural Science, Stellenbosch University, Private Bag X1, Stellenbosch 7602, South Africa; 19547129@sun.ac.za; 2 Postharvest Technology Research Laboratory, Faculty of AgriSciences, Africa Institute for Postharvest Technology, South African Research Chair in Postharvest Technology, Stellenbosch University, Private Bag X1, Stellenbosch 7602, South Africa; tsige@sun.ac.za; 3 Postharvest Research Laboratory, Department of Botany and Plant Biotechnology, University of Johannesburg, P.O. Box 524, Auckland Park, Johannesburg 2006, South Africa

**Keywords:** pomegranate, weight loss, total yeast and mould, antioxidant capacity

## Abstract

The effects of gum arabic (GA; 1.5% *w/v*) and methyl cellulose (MC; 1% *w/v*) enriched with thyme oil (TO; 0.25 and 0.5% *v/v*) on the quality of “Acco” pomegranate arils were studied. Coating treatments, namely, MC, MC + TO_0.5%_, MC + TO_0.25%_, GA, GA + TO_0.5%_ and GA + TO_0.25%_ were applied on arils by dipping, and evaluations were made on physicochemical and microbiological quality, phytochemicals and antioxidant capacity of arils stored (5 ± 1 °C, 95 ± 2% RH) for 16 days. Dipping arils in GA or MC, both containing TO (0.5% *v/v*) significantly (*p* < 0.0001) reduced weight loss and enhanced antioxidant activity (FRAP) (*p* = 0.0014). However, dipping arils in GA combined with TO (0.25% *v/v*) had the highest influence on reducing aril respiration rate compared with other treatments. Overall, results showed that application of coatings (GA + TO_0.5%_ and GA + TO_0.25%_) reduced total yeast and mould and total plate counts and maintained quality up to 8 days of storage. These findings suggest that either GA + TO_0.5%_ or GA + TO_0.25%_ coatings have the capability to extend storage life of “Acco” pomegranate arils.

## 1. Introduction

Pomegranate (*Punica granatum* L.) is an important deciduous shrub belonging to the Lythraceae family, which produces fruit with high amount of polyphenolic compounds, organic acids and minerals [1]. These compounds have several biological activities such as inhibition of oxidation and microbial growth, scavenging reactive oxygen species and reducing the risk of chronic diseases [2,3,4]. Arils, the edible portion, constitute 50% of the fruit (*w/w*) comprising 76–85% juice and 15–24% seed [1,5]. The hard outer rind, which is inedible and often discarded, makes peeling the fruit and separation of arils time-consuming and difficult, limiting the consumption and preference for the fruit [1,3]. An alternative that can increase the consumption of pomegranate whole fruit, which consumers would prefer, is fresh ready-to-eat arils, which offer better convenience and higher commercial value. Nevertheless, arils are susceptible to weight loss, textural and nutritional degradation which limits their shelf life [5]. 

Several postharvest treatments have been applied in order to maintain the quality and extend shelf life of arils which include modified atmosphere packaging (MAP) [6], gamma radiation [7] and controlled atmosphere [8]. However, most of these methods are not entirely effective in reducing postharvest losses during cold storage and shelf life [5].

The adoption of biodegradable edible coatings to preserve postharvest quality of minimally processed products is increasingly becoming a promising option [9]. One possible explanation would be that edible coatings are obtained from natural sources and are therefore biodegradable, and consumers now demand safer and healthier food products with outstanding sensorial attributes. Edible coatings are materials applied in liquid form on fresh produce which suppress water loss and modify internal atmosphere thereby minimizing quality loss [10,11]. Edible coatings like gum arabic and methyl cellulose are commonly used to extend the shelf life of fresh produce [12,13]. Gum arabic is a polysaccharide coating widely used because of good film forming properties, unique emulsification, encapsulating properties and been classified as “generally recognized as safe” (GRAS) by the joint FAO/WHO Expert Committee on Food Additives [14]. Several studies have shown the beneficial effects of the use of gum arabic as an edible coating on green chillies [15], banana [16], tomato [17] and many others in delaying ripening processes, reducing weight loss and microbial growth. Methyl cellulose is polysaccharide derivative of cellulose esters used as the film forming material and formulation of edible coatings. Several studies have shown that application of methyl cellulose can delay ripening, inhibit weight loss and increased intensity of flavour in fresh whole and cut fruit such as banana [18], plums [19] and pomegranate arils [20] among others. 

To improve to antimicrobial properties of edible coatings, essential oils such as lemongrass, thyme, oregano and cinnamon can be incorporated into the polymer matrix [21]. Essential oils inhibit mycelial growth and spore germination of microorganisms which often reduces cellular metabolism and leads to cell death [21,22,23]. Direct application of thyme oil (TO) or combined with edible coatings reportedly reduced decay incidence and microbial proliferation in fresh fruit such as strawberries, avocado and ground beef patties [24,25,26]. When applied on food products, the major active component of TO, thymol, promotes ion leakage from cell membranes, thereby compromising membrane integrity of microorganisms and improves antioxidant system through stimulated biosynthesis of phenolic compounds [21,27]. Furthermore, Valiathan and Athmaselvi [15] demonstrated that TO (0.5%) enriched GA (5%) coatings reduced loss of weight, total soluble solids and colour of coated green chillies stored at 28 °C for 12 days. To our best knowledge, there are no studies reporting the application of GA and MC with or without TO on “Acco” pomegranate arils to preserve postharvest quality and extend shelf life. According to the importance of pomegranate arils as a unique product and limitations with current aril packaging technologies, the objectives of the present study were to determine and characterize GA or MC and essential oil (thyme oil)-based coatings on postharvest quality characteristics of “Acco” pomegranate arils during 16 days of cold storage (5 °C and 95 ± 2% RH) and develop a composite antimicrobial coating.

## 2. Results

### 2.1. Physiological Response

#### 2.1.1. Weight Loss

**Figure 1:**
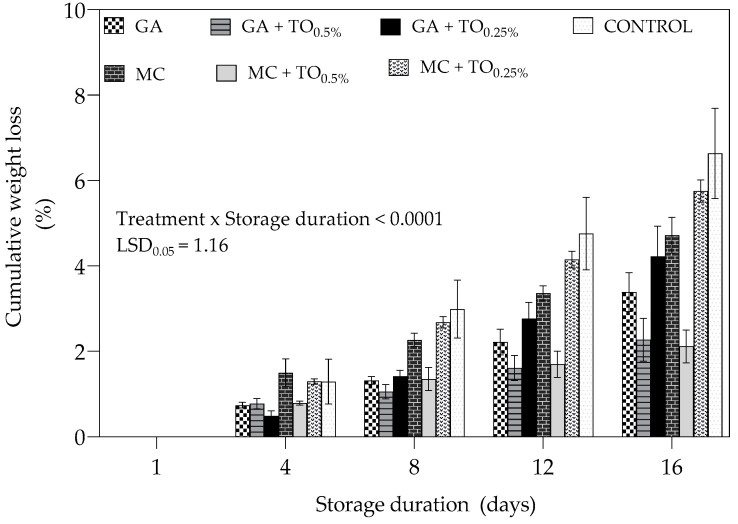
Cumulative weight loss (%) in “Acco” pomegranate arils coated with gum arabic and methyl cellulose coatings enriched with thyme oil (0.5% and 0.25%) and stored at 5 ± 1 °C (95 ± 2% RH) for 16 days. Vertical bars represent the standard error (SE) of mean values of three replicates (1 punnet = 1 replicate). LSD_0.05_ represent least significant difference (*p* < 0.05).

The results presented in Figure 1 show cumulative weight loss of “Acco” pomegranate arils during cold storage period of 16 days. As shown in Figure 1, the change in weight during cold storage demonstrates the efficacy of coating treatments to reduce weight loss compared to uncoated arils. After 16 days, MC + TO_0.5%_ coated and uncoated arils recorded the lowest (2.11%) and highest (6.63%) cumulative weight loss, respectively. 

#### 2.1.2. Respiration Rate

**Figure 2:**
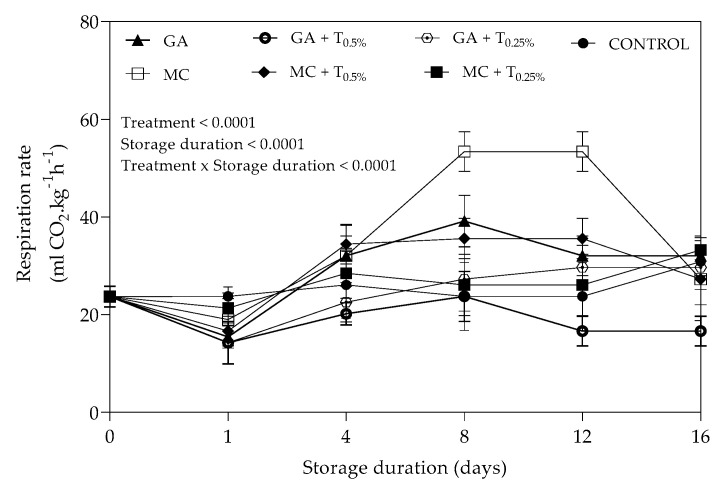
Respiration rate (mlCO_2_ kg^−1^h^−1^) of “Acco” pomegranate arils coated with gum arabic and methyl cellulose coatings enriched with thyme oil (0.5% and 0.25%) and stored at 5 ± 1 °C (95 ± 2% RH) for 16 days. Vertical bars represent the standard error (SE) of mean values of four replicates (1 punnet = 1 replicate). LSD_0.05_ represent least significant difference (*p* < 0.05).

The results showed a significant (*p* = 0.0281) effect of interaction between coating treatments and storage duration on respiration rate (Figure 2). After 16 days of storage, pomegranate arils coated with MC and GA + TO_0.25%_ recorded the highest (53.39 ± 4.05 mlCO_2_ kg^−1^h^−1^) and lowest (16.61 ± 3.06 mlCO_2_ kg^−1^h^−1^) respiration rates, respectively. Application of GA + TO_0.25%_ treatment resulted in the least respiration rate of all coatings and was lower than control on 1, 4, 12 and 16 days of storage. Arils coated with MC, MC + TO_0.25%_, GA and GA + TO_0.25%_ exhibited increase in respiration rates progressively in the beginning of storage followed by substantial decrease in the later stages.

#### 2.1.3. Aril Firmness

**Figure 3:**
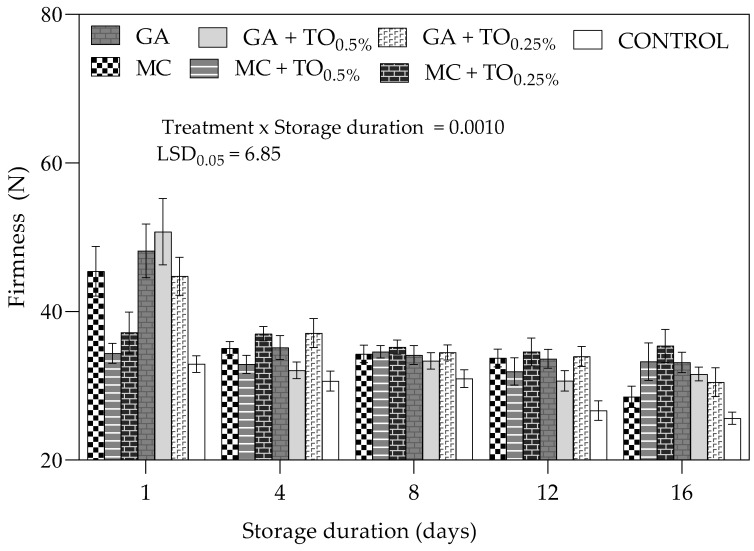
Firmness (N) of “Acco” pomegranate arils coated with gum arabic and methyl cellulose coatings enriched with thyme oil (0.5% and 0.25%) and stored at 5 ± 1 °C (95 ± 2% RH) for 16 days. Vertical bars represent the standard error (SE) of mean values of 3 replicates (1 replicate = 5 arils). LSD_0.05_ represent least significant difference (*p* < 0.05).

Statistical analysis showed that there was a significant (*p* = 0.0010) interaction between coating treatment and storage duration on aril firmness. Figure 3 shows that on day 1, treatment GA + TO_0.5%_ and control recorded the highest (50.75 N) and lowest (32.91 N) aril firmness, respectively. Similarly, on day 16, treatment MC + TO_0.25%_ and control recorded the highest (35.67 N) and lowest (25.64 N) aril firmness, respectively. Overall, there was a decrease in aril firmness across all coating treatments during the entire storage duration. The rate of decrease was more pronounced from day 1 to 4 for coating including MC, GA, GA + TO_0.5%_ and GA + TO_0.25%_.

### 2.2. Physiochemical and Textural Properties

#### 2.2.1. Colour

Table 1 shows change in colour attributes (a*, C and h⁰) of uncoated and coated “Acco” pomegranates during 16 days of storage. For the colour attribute a*, indicating the redness of pomegranate arils, results showed greater positive values (22.70–27.68) on day 1 of analysis, followed by restricted rise until day 8 and decrease until the rest of the storage period. On day 16, methyl cellulose treatments, namely, MC + TO_0.5%_ and MC + TO_0.25%_ retained higher a* values compared to control and GA treatments. For chroma, representing purity or intensity of a colour, there were minimal changes for coated arils, throughout the storage compared to untreated control which significantly decreased until day 16. Moreover, when compared, arils coated with MC + TO_0.5%_ maintained higher chroma values with than untreated control. At day 16, arils coated with GA + TO_0.25%_ recorded the lowest chroma value compared to coated arils and control. The treatments GA + TO_0.5%_ and GA + TO_0.25%_ resulted in significantly lower h⁰ values compared to control.

#### 2.2.2. Titratable Acidity, Total Soluble Solids and TSS/TA

**Figure 4:**
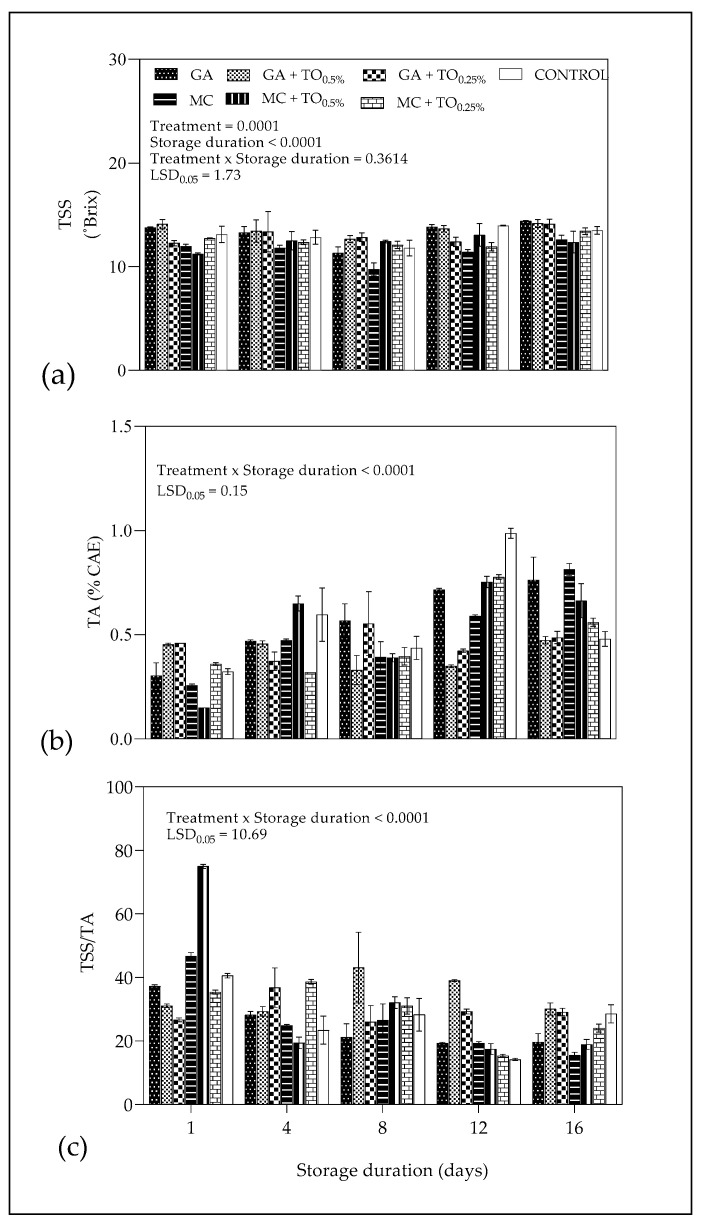
(**a**) Total soluble solids (⁰Brix), (**b**) titratable acidity (% citric acid equivalents) and (**c**) TSS/TA ratio of “Acco” pomegranate arils coated with gum arabic and methyl cellulose coatings enriched with thyme oil (0.5% and 0.25%) and stored at 5 ± 1 °C (95 ± 2% RH) for 16 days. Vertical bars represent the standard error (SE) of mean values of three replicates. LSD_0.05_ represent least significant difference (*p* < 0.05).

Figure 4a shows a significant (*p* < 0.0001) effect of main effects coating treatments and storage duration on TSS of aril juice during cold storage. Titratable acidity values ranged from 0.15 to 0.46% on day 1 for juice extracted from coated arils and showed a fluctuating increase with passage of storage. On day 16, the highest TA content was recorded for juice extracted from GA + TO_0.25%_ (0.81%) coated arils, while the lowest acidity values corresponded to MC (0.47%)-coated arils (Figure 4b). On day 16, GA + TO_0.5%_ (14.67) and MC (12.6) treatments recorded the highest and lowest TSS for coated arils. There was a significant (*p* < 0.001) interaction between storage duration and coating treatment on TSS/TA (Figure 4c). On day 1 of storage, MC + TO_0.5%_ and GA + TO_0.25%_ recorded the highest (74.89 ± 0.59) and lowest (26.67 ± 0.59) TSS/TA values, respectively. However, at the end of cold storage, TSS/TA value for MC + TO_0.5%_, was comparatively lower than GA + TO_0.25%_ and uncoated control samples (30.08 and 28.54, respectively). 

### 2.3. Microbial Quality

There was a significant (*p* < 0.0001) interaction between storage duration and coating treatments on YM growth and TPC (total plate counts)/aerobic bacteria (Table 2). The results showed an increasing tendency of YM growth and TPC in all samples throughout the storage period of 12 days. On day 1, arils coated with MC and GA + TO_0.5%_ recorded lowest (2.80 ± 0.01) TPC, significantly lower than uncoated control. However, on day 16, GA + TO_0.25%_ coated arils recorded the lease TP counts, significantly lower that all coating treatment and control. The rate of increase in YM growth was highest in MC-treated arils followed by untreated control for all the sampling days. Moreover, YM growth was markedly inhibited by dipping arils in GA + TO_0.5%_ and GA + TO_0.25%_ coatings. The aerobic bacteria reached maximum of 4.53 log CFU g^−1^ after 4 days of cold storage. However, for GA + TO_0.25%_-coated arils, aerobic bacteria values were lower than the maximum acceptable limit of 7 log CFU g^−1^. In this research work, higher concentration of TO (0.5% *v/v*) was effective to reduce YM growth when used with GA and not MC coating as the carrier. The same observation applied for aerobic bacteria, except that lower concentration of TO (0.25%) retained higher antimicrobial properties as storage duration was extended.

### 2.4. Phytochemical and Antioxidant Content

#### 2.4.1. Phytochemical Content

**Figure 5:**
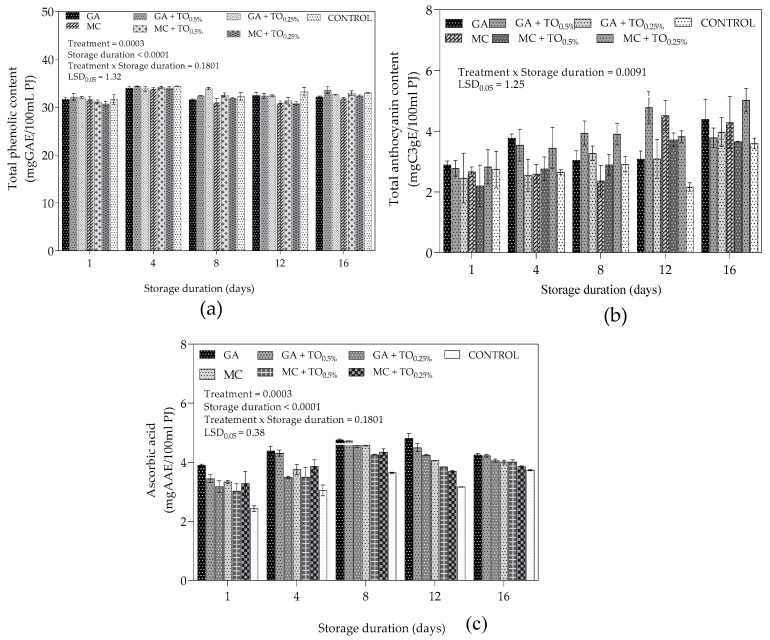
(**a**) Total phenolic content (mgGAE/100 mL PJ), (**b**) total anthocyanin content (mgGAE/100 mL PJ) and (**c**) ascorbic acid (mgAAE/100 mL PJ) “Acco” pomegranate arils coated with gum arabic and methyl cellulose coatings enriched with thyme oil (0.5% and 0.25%) and stored at 5 ± 1 °C (95 ± 2% RH) for 16 days. Error bars represent standard error (SE) of the mean values ± S.E. of three replicates (1 punnet = 1 replicate). LSD_0.05_ represent least significant difference (*p* < 0.05).

The evolution of total phenolic (TP) content of coated and uncoated “Acco” pomegranate arils during cold storage is shown Figure 5. The graph shows that TP content initially increased from day 1 to 4, for both coated and uncoated arils. From day 4 to 8, there was a sharp decrease in TP for all treatments except GA + TO_0.25%_ coated arils. The rate of decrease in TP content was greater in coated arils except for GA + TO_0.5%_ when compared to uncoated arils. The results show a significant effect of interaction between treatment and storage duration on total anthocyanin content (TAC) (*p* = 0.0091) (Figure 5). On day 1, treatments MC and GA + TO_0.5%_ recorded highest (2.89 ± 0.13 mgC3E/100 mL PJ) and lowest (2.20 ± 0.69 mgC3E/100 mL PJ) TAC, respectively. However, on day 16, GA + TO_0.5%_ and control had the highest (5.03 ± 0.37 mgC3E/100 mL PJ) and lowest (3.60 ± 0.17 mgC3E/100 mL PJ) TAC, respectively. During storage, evolution of TAC did not follow a distinct pattern, however, TAC for GA + TO_0.5%_-, GA + TO_0.25%_- and MC + TO_0.25%_-treated arils mostly increased from day 1 to 16 total anthocyanin content. Main effects namely coating treatment (*p* = 0.003) and storage duration (*p* < 0.0004) had significant effect on ascorbic acid (AA) content. Figure 4 shows that coated arils had higher AA retention compared to uncoated arils. Initially, AA content increased from day 1 to 8 followed by a steady decrease till day 16 of storage. The decreasing pattern was much more pronounced in uncoated arils when compared to coated arils. 

#### 2.4.2. Antioxidant Content

**Figure 6:**
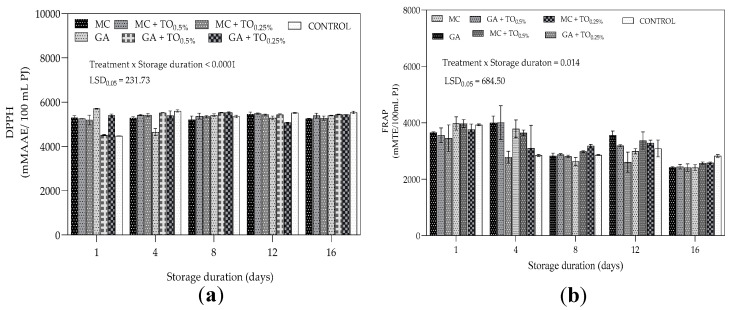
(**a**) DPPH (mMAAE/100 mL PJ) and (**b**) FRAP (mMAAE/100 mL PJ) of “Acco” pomegranate arils coated with gum arabic and methyl cellulose coatings enriched with thyme oil (0.5% and 0.25%) and stored at 5 ± 1 °C (95 ± 2% RH) for 16 days. Error bars represent standard error (SE) of the mean values ± S.E. of three replicates. LSD_0.05_ represent least significant difference (*p* < 0.05).

Antioxidant capacity of pomegranate juice was measured using DPPH radical scavenging activity (DPPH) and ferric reducing antioxidant power (FRAP) and results are presented in Figure 6. The DPPH and FRAP assays showed differences among determinations of antioxidant capacity. There was a significant interaction of storage duration and coating treatment for both radical scavenging activity (*p* < 0.0001) (Figure 6a) and reducing antioxidant power (*p* = 0.0014) (Figure 6b). On day 1, GA + TO_0.5%_ treatment and control recorded comparatively higher radical scavenging activity compared to all coated treatments. Application of coating treatments MC + TO_0.5%_, MC + TO_0.25%_, GA + TO_0.25%_ and MC had no significant effect on antioxidant potential of arils from day 1 to 16 of cold storage. Gum arabic (GA) coatings initially increased antioxidant potential of arils between day 1 and 4; however, DPPH values decreased from day 4 to 8 and had restricted change with extension of storage. Despite fluctuations from day 1 to 12, coating treatment MC + TO_0.5%_, maintained higher reducing antioxidant power compared to uncoated arils. Overall, relative to FRAP values on day 1, there was decrease in antioxidant potential across all treatments, observed from day 1 until end of storage. Treatments MC, MC + TO_0.5%_, MC + TO_0.25%_ and GA recorded markedly higher FRAP values compared to untreated control for most periods during storage. 

## 3. Discussion

### 3.1. Physiological Response

#### 3.1.1. Weight Loss

In this study, both MC and GA coating treatments effectively reduced weight loss by creating a physical barrier to water loss compared to control [28,29]. Methyl cellulose and gum arabic coatings are both polysaccharides of hydrophobic nature which reduce penetration of water and oxygen when applied [14,18]. Elevated weight loss in uncoated arils could be explained by increased metabolic activity associated with tissue senescence [30]. Application of coatings on arils maintained weight loss either below or within acceptable limit (4–6%) for minimally processed fruit for the storage period of 16 days [31].

#### 3.1.2. Respiration Rate

Rapid decrease in respiration rate of arils from harvest (day 0) to day 1 can be explained by initial response to cold storage temperature (5 °C); marked reduction in respiration rate at lower storage temperature has been reported for pomegranate arils and other fresh produce [32,33]. Yousuf and Srivastava [34] observed lower CO_2_ production for arils coated with flaxseed gum (0.3 and 0.6%) enriched with lemongrass oil (0–800 ppm) when compared to uncoated arils. The results were indirectly related to inhibitory effects of flaxseed gum on respiration rate for all coated arils. Similarly, application of coatings such as *Aloe vera* gel with antimicrobial properties and starch on pomegranate arils resulted in lower respiration rate [35,36]. The findings corroborate with the authors of [17], who observed reduced respiration rate in tomato coated with different concentrations of GA (5, 10 and 20%). Likewise, the respiration rate pattern for arils coated with MC, MC + TO_0.25%_, GA and GA + TO_0.25%_ in this study resembles observations by [37] for cherries coated with chitosan acetate at different concentration (1, 3, 5, 10 and 20 g/L) during 20 days of cold storage period (4 °C). In this study, higher respiration rates recorded for coating treatments such as MC, MC + TO_0.25%_ and GA between day 1 and 12 be an indication of deterioration of aril quality induced by spoilage microorganisms growing under anaerobic conditions. This was confirmed by the results for microbial growth reported in Section 2.3.

#### 3.1.3. Aril Firmness

The general decrease in aril firmness observed across all coating treatments can be explained by several factors such as increase in microbial load [38], moisture loss [39], senescence [40] and enzymatic cell membrane deterioration [41]. Coating treatments (GA + TO_0.5%_, GA + TO_0.25%_ and MC + TO_0.25%_) maintained higher aril firmness than uncoated control throughout the storage duration, with significant difference observed on day 1 and 16. Valero and Serrano [41] reported that application of coatings on fresh produce inhibits softening by minimising loss of important cell wall pectins and activity of the cell wall hydrolases. Similarly, Martínez-Romero [35] observed that application of *Aloe vera* gel (50 or 100%) as a coating on pomegranate arils resulted in significant delay in aril softening during storage at 3 °C. 

### 3.2. Physiochemical and Textural Properties

#### 3.2.1. Colour

Change in aril colour was not clear; however, application of MC + TO_0.5%_ and MC + TO_0.25%_ coatings inhibits the loss of redness compared to uncoated fruit arils. Likewise, Öz and Eker [42] reported reduced loss of redness in arils extracted from “Tarom” pomegranates treated with starch coatings enriched with of *N. sativa* before cold storage (4 °C and 95% RH) for 12 days. The observed changes in chroma values corroborate with Azarakhsh et al. [43] who observed a decrease over time in chroma values of fresh-cut pineapple coated with gellan gum-based coating during cold storage. Yousuf and Srivastava [34] similarly observed decrease in chroma values with passage of storage for pomegranate arils coated with flaxseed gum for 12 days storage period at 5 °C. Lower hue angle (h⁰) values are associated with the degradation of red colour of pomegranate arils [44,45]. Lower h⁰ values recorded for GA + TO_0.5%_ and GA + TO_0.25%_ compared to control can be explained by the notably higher opacity that inhibits light absorption on the coated aril surface, thus reducing its red colour [31].

#### 3.2.2. Titratable Acidity, Total Soluble Solids and TSS/TA

The results for TSS evolution in coated arils were in conformity with [46] who similarly observed reduced increase in TSS for chitosan coated plum during 35 days of cold storage (1 °C). In addition, research findings by Maftoonazad and Ramaswamy [18] reported reduced TSS increase for “Berangan” banana stored at 26 °C coated with methyl cellulose, sodium carboxymethyl cellulose and hydroxypropylmethyl cellulose coating. For TA, results corroborate with Yousuf and Srivastava [34], who observed an increase in TA for pomegranate arils coated with flaxseed gum containing lemongrass oil during 12 days of cold storage (5 °C). Rapid decrease in TA is associated with faster senescence, and therefore edible coatings as surface barriers modify the internal atmosphere of the fruit to prevent the decrease in TA content through acid metabolism [47]. The pattern for TSS/TA is not clear and often fluctuates for most coating treatments. Previous studies show that coatings inhibit ripening process during storage which reduces increase in TSS/TA for whole and minimally processed fruit including banana [48] and sweet cherries [37].

### 3.3. Microbial Quality

The increase in YM growth of arils coated with GA + TO_0.5%_ treatment after 12 days, were below the maximum limit of 5 log CFU g^−1^ allowed in raw and fresh-cut fruit allowed by the South African legislation [49]. Gum arabic structure consist of arabinogalactan oligosaccharides, polysaccharides and glycoproteins which form a semipermeable barrier to limit microbial growth on food products [50]. Sellamuthu et al. [22] found main components of TO as phenolic monoterpene thymol (58.77%), terpene hydrocarbon with an aromatic ring (4-isopropyltoluene) and cymol (17.82%). Thyme oil is hydrophobic which enables it to permeate the lipid component of the microbial cell membrane and mitochondria, disturbing the pathogen cell structures causing cell death [26,27]. The marked increase in microbial growth particularly for arils coated with MC treatments suggests that while coatings form a barrier between aril and microbes, they may provide a conducive environment for microbial proliferation.

### 3.4. Phytochemical and Antioxidant Content

#### 3.4.1. Phytochemical Content

Uncoated arils recorded higher TP content compared to MC (MC, MC + TO_0.5%_ and MC + TO_0.25%_)- and GA (GA + TO_0.5%_ and GA + TO_0.25%_)-coated arils for most periods during storage. The results were contrary to Saba and Amini [20], who reported higher decrease in TP content for uncoated arils compared to MC coated arils. Similarly, Ghasemnezhad et al. [51] observed a decrease in TP content of “Tarom” pomegranate arils stored at 4 °C for 12 days. Polyphenoloxidase and peroxidase oxidation of phenolics during cold storage during post-harvest storage has been ascribed as the main factor decrease in TP content [52,53,54]. Application of treatments GA + TO_0.5%_ appeared to delay the degradation of TP content during storage similar to chitosan coatings reported by Ghasemnezhad et al. [51]. Active compounds in TO, such as *β*-linalool, thymol and caryophyllene, have antibrowning properties could have delayed the oxidative reaction when incorporate in GA coatings [38,39,40]. 

The observed increase in TAC for GA + TO_0.5%_, GA + TO_0.25%_ and MC + TO_0.25%_ could be attributed to continuation of anthocyanin synthesis during the cold storage, similarly observed by Varasteh et al. [45] for chitosan coated “Rabbab-e-Neyriz” pomegranates. Several studies have associated the change in composition of anthocyanin content during cold storage with the activity of the anthocyanin biosynthetic pathway enzymes [55,56,57]. Earlier studies by Gil et al. [58] report that anthocyanin concentration increased during cold storage. However, recently, Varasteh et al. [45] showed that TAC of “Rabbab-e-Neyriz” pomegranate juice decreased during storage at either 2 or 5 °C. In the same study, application of chitosan coatings inhibited loss of anthocyanins during storage. The findings by Varasteh et al. [45] interrelate with our results demonstrating GA, GA + TO_0.5%_, GA + TO_0.25%_ and MC + TO_0.5%_ minimised the degradation of anthocyanins and improved their biosynthesis. Reduced decline and higher AA content recorded for both MC (MC, MC + TO_0.5%_ and MC + TO_0.25%_)- and GA (GA + TO_0.5%_ and GA + TO_0.25%_)-coated arils was similarly reported by Barman et al. [59] for putrescine and carnauba wax coated arils. After day 8, the decline of AA content could be an indication of increased rate of senescence associated with loss of cell integrity AA content [60]. Studies have shown that edible coatings modify internal atmosphere of fresh produce which promotes accumulation of secondary metabolites and AA during cold storage [61].

#### 3.4.2. Antioxidant Content

The general decrease in antioxidant capacity (DPPH) observed across all treatments for GA could be explained by possible senescence and decay which often reduces antioxidant capacity [62]. For the DPPH assay, the difference in antioxidant capacity between coated and uncoated arils was not clear. Contrary to the present results, Nair et al. [63] showed that guava fruit coated with chitosan and alginate coatings recorded higher antioxidant potential compared to uncoated fruit. Similarly, Wang and Gao [62] demonstrated that application of chitosan-based coatings at different concentrations (0.5, 1.0 and 1.5%) on strawberry during cold storage (5 or 10 °C) resulted in higher antioxidant activity compared to uncoated fruit. The results from FRAP assay showed suggests that application of GA and MC treatments with thyme oil (0.5 or 0.25%) reduced loss of antioxidant potential (FRAP) of arils during storage. A possible explanation is the production of secondary metabolites and ascorbic acid under modified atmosphere induced by surface coatings, which improves antioxidant capacity [62]. Furthermore, modified internal atmosphere in coated fruit has been attributed to reduced metabolism and synthesis of phenolics and flavonoids [64] The conditions promote the accumulation of secondary metabolites and ascorbic acid which increases antioxidant capacity [65].

## 4. Materials and Methods

### 4.1. Fruit Supply

Pomegranate (cv. Acco) fruit were harvested at commercial maturity (TSS =1.63 ± 0.12 ⁰Brix, pH = 3, % Citric acid = 1.16 ± 0.17) from Blydeverwacht Farm, Wellington, Western Cape (33°48′0″ S, 19°53′0″ E), South Africa. Healthy fruit (fruit without defects) were phytosanitised by washing in commercial pomegranate fungicide (TeacherTM solution at 600 ppm) for 3 min and allowing fruit to dry at room temperature prior to aril extraction.

### 4.2. Preparation of Raw Materials and Experimental Layout

#### 4.2.1. Preparation of Coating Solutions

Methyl cellulose (1% *w/v*) coating solution was formulated following the procedure outlined by Maftoonazad and Ramaswamy [28] with some slight changes. Briefly, methyl cellulose (1 g) powder was dissolved in a 100 mL water–ethyl alcohol mixture (31:11) at 90 °C under magnetic stirring for 45 min. Following that, glycerol (1%) was added to the solution as a plasticiser followed by tween 80 (0.05%) and either 0.5 or 0.25% of thyme oil (TO). The coating solution was homogenised at 2500 r.p.m. for 30 min in an overhead stirrer (Separation Scientific, Johannesburg, South Africa). The three methyl cellulose treatments were MC, MC + TO_0.5%_ and MC + TO_0.25%_. Gum arabic (1.5%) solution was prepared according to the method reported by [31] with some slight modifications. Briefly, 1.5 g of gum arabic was dissolved in 100 mL distilled water and continuously stirred at low heat (50 °C) for 90 min on a hot plate stirrer. The coating solution was then filtered using cheese cloth to remove any undissolved impurities and allowed to cool to 20 °C before addition of glycerol (1%). Tween 80 (0.05%) was added to the formulation followed by either 0.5 or 0.25% of thyme oil. Last, the pH of the coating solution was adjusted to 5.6 with NaOH solution. The final coating solution was homogenised at 2500 r.p.m. for 30 min in an overhead stirrer (Separation Scientific, Johannesburg, South Africa). The three gum arabic treatments were GA, GA + TO_0.5%_ and GA + TO_0.25%_. 

#### 4.2.2. Aril Processing 

Phytosanitised fruit were manually peeled using sharp knives (Sigma-Aldrich, Johannesburg, South Africa) with extensive care wearing polythene hand gloves (Sigma-Aldrich, Johannesburg, South Africa) and face masks (Sigma-Aldrich, Johannesburg, South Africa) to avoid any contamination. Extracted arils were combined and well mixed to ensure uniformity in large sterilized buckets for later use. 

#### 4.2.3. Experimental Layout

A completely randomized design was used. Arils were divided into seven groups representing each treatment combination and control. For each sampling day, a group of arils weighing 900 g was further divided into three clustered group weighing 300 g each. Baseline measurements for microbial and physicochemical properties of fresh arils were determined before packaging on day 0. Application of coating treatments (MC, MC + TO_0.5%,_ MC + TO_0.25%_, GA, GA + TO_0.5%_ and GA + TO_0.25%_) was done by dipping arils for 1 min into solutions, thereafter drained with a colander (Sigma-Aldrich, Johannesburg, South Africa) and collected on a tray before aril drying at room temperature for 45 min. Aril portions of 150 g in each cluster were packaged into 180 mL tub and lid polyethylene terephthalate punnets (Zibo plastics, Kuils River, South Africa) previously sterilised with ethanol (70%). Packaged samples were stored at 5 °C and 95 ± 2% RH for 16 days, and sampling was carried out on 0, 1, 4, 8, 12 and 16 days of storage. 

### 4.3. Physiological Response

#### 4.3.1. Weight Loss

The weight of each aril packaging was determined using an electronic weighing balance (ML3002.E, Mettler Toledo, Switzerland) [6]. Weight loss was determined using the following equation,

WL = (W_o_ − W_f_)/Wo × 100) (1)

where WL represents weight loss (%), W_o_ is the initial weight (g) of punnet and W_f_ is the final weight (g) prior to punnet analysis. Three punnets (150 g of arils each) per treatment and control were weighed on each sampling day and results were expressed as mean ± standard error (S.E.).

#### 4.3.2. Headspace Gas Composition

The gas composition (oxygen and carbon dioxide) inside the punnets was determined using a gas analyser with an accuracy of 0.5% (Checkmate 3, PBI Dansensor, Ringstead, Denmark) before opening them for other quality measurements on sampling days [6]. Gas analysis was performed by inserting the needle attached to the gas analyser through the lid of punnet in triplicates for each treatment. The device was auto calibrated with the atmospheric gas composition and the results were expressed as percentage of gases. Four punnets (150 g of arils each) per treatment and control were evaluated on each sampling day and results were expressed as mean ± S.E.

### 4.4. Physico-Chemical and Textural Properties

#### 4.4.1. Colour

Pomegranate aril colour was determined in CIELAB coordinates (L*, a*, b*) using a Minolta Chroma Meter CR-400 (Minolta Corp., Osaka, Japan) as reported by Caleb et al. [66]. The colour parameters chroma (C* = (a*^2^ + b*^2^)^1/2^) and hue angle [h⁰ = arctan (b*/a*)] were calculated. The mean of 10 measurements were calculated for each replication per treatment. Three punnets (150 g of arils each) per treatment and control were evaluated and results were expressed as mean ± S.E. per interval.

#### 4.4.2. Aril Firmness

The firmness of arils was determined using texture analyser (TA-XT Plus, Stable Micro Systems, Surrey, England), with a 35 mm diameter cylindrical probe [67]. The maximum compression force (N) required to rupture arils at a test speed of 1.0 mms^−1^ and distance of 9.5 mm were used to evaluate firmness. Three punnets (150 g of arils each) per treatment and control were evaluated, and a total of 15 arils obtained from each punnet were measured individually and results were expressed as mean ± S.E. per interval.

#### 4.4.3. Titratable Acidity and Total Soluble Solids

Pomegranate juice from arils packaged in each punnet (150 g) were extracted using a blender (Mellerware, South Africa) and directly used for measuring titratable acidity (TA), total soluble solids (TSS) and pH [68]. Measurements of TA were done by titration method to an end point of pH 8.2 using a Metrohm 862 compact titrosampler (Herisau, Switzerland) and the results were expressed as percentage citric acid. Total soluble solids (TSS) were determined using a digital refractometer (Atago, Tokyo, Japan). TSS/TA ratio was calculated to understand further the relationship between TSS and TA. 

### 4.5. Microbial Quality

Microbiological quality of aril samples was analysed for aerobic mesophilic bacteria, yeasts and moulds by pour-plate method [69], using total plate count agar (FAA, Lab M, Bury, Lancs, UK) and chloramphenicol agar (Biolab, Merck, Kenilworth, NJ, USA), respectively. To measure microbial load, ten grams of arils per punnet was taken aseptically and blended with 40 mL of sterile physiological solution. A dilution series was made to 10^−4^ and from each dilution, 1 mL of was used to prepare pour plates for the total plate count and spread plates for the yeast and moulds. Each dilution was plated out in triplicate. Plates were incubated at 37 C for 2 days for aerobic mesophilic bacteria, and at 26 °C for 48 h for yeast and moulds. After 48 h of incubation, the plates with a range of 30 to 300 colonies were counted. The results were reported as log CFU mL^−1^ [69].

### 4.6. Phytochemical and Antioxidant Content

#### 4.6.1. Phytochemical Content

Total phenolic content was determined according to the Folin–Ciocalteu colorimetric method [69]. Gallic acid was used as standard for calibration curve, and TPC was expressed as milligram Gallic acid equivalent (GAE) per 100 mL of pomegranate juice (PJ) (mg/100 mL). Briefly, in triplicates, diluted juice extract (50 µL) was mixed with 450 µL of 50% methanol followed by the addition of 500 µL Folin–C and then sodium carbonate (2%) solution after 2 min. The mixture was vortexed and absorbance read at 725 nm using a UV–visible spectrophotometer (Thermo Scientific Technologies, Madison, Wisconsin). The pH differential method was used to determine total monomeric anthocyanin concentration using two buffer systems namely potassium chloride buffer for pH, 10.0 (0.0025 M) and sodium acetate buffer for pH, 4.5 (0.4 M) [70]. Briefly, in triplicates, supernatant (1 mL) from extracted juice was diluted with 7 mL of potassium chloride buffer (pH 1.0) and sodium acetate buffer (pH 4.5), separately and results determined at 510 nm and 700 nm in a UV–Visible spectrophotometer (Thermo Scientific Technologies, Madison, Wisconsin). Duplicate readings were done for the triplicate arils juice samples. Results were expressed as milligram cyanidin-3-glucoside equivalent per 100 mL pomegranate juice (mgC3gE/100 mL PJ) according to Equations (3) and (4).

A = (A500 − A770)pH1.0 − (A510 − A700)pH4.5(2)

TAC (mg100 mL^−1^) = [(A × MW × DF × 100)/Є] × L (3)

where A = Absorbance values at 510 nm and 700 nm, Є = Cyanidin-3-glucoside molar absorbance (26,900), MW = Cyanidin-3-glucoside molecular weight (449.2 g/mol), DF = Dilution factor, L = Cell path length (1 cm) and TAC = total anthocyanin content.

Ascorbic acid was measured spectrophotometrically according to Atukuri et al. [71] with slight modifications. In triplicates, 1 mL of pomegranate juice was mixed with 14 mL of 1% Metaphosphoric acid followed by sonication on ice for 4 min and centrifugation at 4000× *g* for 12 min. Following that, 1 mL of the supernatant was pipetted into a tube and mixed with 9 mL of 2, 6 dichlorophenolindophenol dye (0.0025%). After a period of incubation in the dark (10 min), the samples read 515 nm wavelength. Ascorbic acid values were extrapolated from a standard curve (0.01–0.12 mg/mL) with R^2^ > 0.90 and results were expressed as ascorbic acid units per 100 millilitre crude juice (mgAAE/100 mL PJ).

#### 4.6.2. Antioxidant Content

Determination of free radical scavenging activity was determined spectrophotometrically according to the procedure described by Fawole et al. [70]. In triplicates, 15 μL of PJ was first diluted with methanol (735 μL) after which DPPH solution (0.1 mM, 750 μL) was added to the mixture. After vortexing followed by 30 min of incubation in the dark, determination of sample absorbance was made at 517 nm. Free radical-scavenging activity of juice was expressed as ascorbic acid (millimoles) equivalent per 100 millilitres of crude juice (mM AAE/100 mL PJ). Ferric reducing antioxidant power assay was evaluated using FRAP solution prepared from 25 mL acetate buffer (300 mM acetate buffer, pH 3.6), 132 2.5 mL (10 mM of TPTZ solution) and 2.5 mL (20 mM of FeCl_3_ solution) [70]. Ten millilitres of aqueous methanol (50%) was added to juice (1 mL) in triplicates, sonicated for 10 min and centrifuged for 10 min at 4000 rpm. Following that, the solutions were mixed with 2850 µL FRAP and measurements were made at 593 nm spectrophotometrically after 30 min incubation. Trolox (100–1000 µM) standard curve was used to extrapolate values and results were expressed as trolox (mM) equivalents per millilitre pomegranate juice (mM TE/100 mL PJ).

### 4.7. Statistical Analysis

All experimental data obtained were subjected to two-way analysis of variance (ANOVA) at 95% confidence interval using SAS Software (SAS Enterprise Guideline 7.1, Carrey, NC, USA). The post hoc test (Duncan’s Multiple Range Test) was applied to determine the least significant differences (LSD) among means at 5% significance level.

## 5. Conclusions

The results of this study indicate that coatings formulated from gum arabic or methyl cellulose combined with either 0.5 or 0.25% of thyme oil are an effective way of maintaining the physicochemical, some microbiological and biochemical qualities of “Acco” pomegranate arils. Coatings reduced weight loss and preserved total soluble solids and titratable acidity content. Gum arabic coatings proved to be a better option as carrier of thyme oil to reduce microbial proliferation. Moreover, gum arabic coatings significantly reduced microorganism counts compared to uncoated control, resulting in a better maintenance of safety of the arils up to 12 days of cold storage at 5 °C. Overall, based on the microbial quality tested, the maximum storability period of arils could be 8 days when coated with gum arabic combined with 0.5% *v*/*v* of thyme oil when compared to uncoated arils which had a shelf life less than 4 days. Application of MC treatments with thyme oil (0.5%) improved the antioxidant potential but did not improve the microbial quality of arils. However, the smell of thyme oil was very strong during storage and easily observed at low intensity on day 16. Therefore, a sensory evaluation study would be recommended to determine the consumer preference of coated arils.

## Figures and Tables

**Table 1:** Effects of gum arabic and methyl cellulose coatings enriched with thyme oil (0.5% and 0.25%) on colour attributes reported as redness (a*), chroma (C*) and hue angle (h⁰) of “Acco” pomegranate arils during storage at 5 °C for 16 days.

				Storage Duration (days)			
Colour Attribute	Treatment	0	1	4	8	12	16
	Control	25.41	27.68 ±0.67ab	24.96 ± 0.16c–i	24.83 ± 0.32c–i	23.93 ± 0.54e–m	24.65 ± 0.18c–l
a*	GA		23.97 ± 0.37e–m	25.28 ± 1.10c–h	25.44 ± 0.85c–g	24.09 ± 0.52e–m	22.60 ± 0.37lm
	GA + TO_0.5%_		22.70 ± 0.29klm	22.93 ± 0.46i–m	23.95 ± 0.44e–m	23.68 ± 0.19f–m	24.30 ± 0.40d–l
	GA + TO_0.25%_		22.99 ± 0.72i–m	24.44 ± 0.79c–l	24.26 ± 0.73d–m	23.13 ± 0.54i–m	23.50 ± 0.74g–m
	MC		24.77 ± 0.55c–j	22.23 ± 0.85m	23.24 ± 0.60h–i	26.25 ± 1.05a–d	22.76 ± 0.55j–m
	MC + TO_0.5%_		25.73 ± 0.34c–f	27.80 ± 0.33a	26.19 ± 1.07a–d	24.82 ± 0.21c–j	24.72 ± 0.82c–k
	MC + TO_0.25%_		25.25 ± 0.17c–h	26.40 ± 0.15abc	23.43 ± 0.57g–m	25.87 ± 0.26b–e	24.75 ± 0.46c–k
C*	Control	27.56	22.22 ± 0.51f–k	23.38 ± 0.91b–g	23.72 ± 0.77b–f	22.33 ± 0.46e–k	21.05 ± 0.33jk
	GA		21.28 ± 0.26ijk	22.00 ± 0.09f–k	22.58 ± 0.37d–k	22.54 ± 0.12d–k	22.72 ± 0.48c–j
	GA + TO_0.5%_		21.59 ± 0.64h–k	23.04 ± 0.61b–h	22.92 ± 0.55c–i	21.88 ± 0.46g–k	22.19 ± 0.58f–k
	GA + TO_0.25%_		23.29 ± 0.57b–h	20.85 ± 0.83k	21.68 ± 0.49g–k	24.48 ± 0.90abc	21.26 ± 0.43ijk
	MC		24.22 ± 0.31a–d	25.61 ± 0.08a	24.20 ± 0.69a–d	23.19 ± 0.28b–h	23.09 ± 0.85b–h
	MC + TO_0.5%_		24.05 ± 0.02a–e	24.68 ± 0.09ab	22.22 ± 0.46f–k	24.25 ± 0.21a–d	23.33 ± 0.43b–h
	MC + TO_0.25%_		25.60 ± 0.59a	23.14 ± 0.25b–h	23.20 ± 0.15b–h	22.39 ± 0.54e–k	23.07 ± 0.17b–h
	Baseline		22.15				
	Control		21.28 ± 0.38a				
	GA		21.59 ± 0.38a				
	GA + TO_0.5%_		19.57 ± 0.42c				
h⁰	GA + TO_0.25%_		19.13 ± 0.39c				
	MC		20.71 ± 0.23ab				
	MC + TO_0.5%_		21.23 ± 0.47a				
	MC + TO_0.25%_		19.80 ± 0.25bc				
	Level of significance		Coating treatment (A)	Storage duration (B)	A × B		
	a*		<0.0001	0.0222	<0.0001		
	C*		<0.0001	0.0368	<0.0001		
	h⁰		<0.0001	0.3498	0.2729		

All values are presented as mean ± SE. Means presented in the same column with different letters indicate significant differences between impacts (*p* < 0.05). Means presented in the same row with different letters indicate significant differences between storage duration (*p* < 0.05), according to Duncan’s multiple range test.

**Table 2:** Effects of gum arabic and methyl cellulose coating emulsions containing thyme oil (0.5% and 0.25%) on yeast and mould counts (log CFU g^−1^) and total plate counts (log CFU g^−1^) of “Acco” pomegranate arils stored at 5 ± 1 °C (95 ± 2% RH) for 16 days.

			Storage Duration (Days)			
Microbial Quality	Coating Treatment	0	1	4	8	12
	MC		4.05 ± 0.01def	TMTC	TMTC	TMTC
	MC + TO_0.5%_		3.78 ± 0.11e–h	4.97 ± 0.04bc	5.31 ± 0.04a	TMTC
Yeast and mould counts (log CFU g^−1^)	MC + TO_0.25%_		TFTC	4.70 ± 0.01c	4.15 ± 0.01d	TMTC
	GA		3.67 ± 0.01h	5.30 ± 0.00a	5.29 ± 0.05ab	TMTC
	GA + TO_0.5%_		TFTC	3.22 ± 0.02i	3.70 ± 0.14gh	4.05 ± 0.15de
	GA + TO_0.25%_		TFTC	3.73 ± 0.01fgh	TMTC	TMTC
	CONTROL	3.98	3.90 ± 0.02d–g	4.01 ± 0.01d-g	TMTC	4.05 ± 0.01def
Total plate counts (log CFU g^−1^)	MC		5.87 ± 0.01c	TMTC	TMTC	TMTC
	MC + TO_0.5%_		TMTC	TMTC	TMTC	TMTC
	MC + TO_0.25%_		3.09 ± 0.02h	4.99 ± 0.05d	6.22 ± 0.01ab	TMTC
	GA		TMTC	6.43 ± 0.00a	TMTC	TMTC
	GA + TO_0.5%_		2.80 ± 0.01i	4.53 ± 0.02e	6.22 ± 0.01ab	TMTC
	GA + TO_0.25%_		3.38 ± 0.00g	4.54 ± 0.03e	5.85 ± 0.04c	6.11 ± 0.13bc
	CONTROL	4.21	4.08 ± 0.08f	TMTC	TMTC	5.83 ± 0.11c
Level of significance	Coating treatment × Storage duration					
Yeast and mould counts	<0.0001					
Total plate counts	<0.0001					

All values are presented as mean ± SE. Means presented in the same column with different letters indicate significant differences between coating treatments (*p* < 0.05). Means presented in the same row with different letters indicate significant differences between storage duration (*p* < 0.05), according to Duncan’s multiple range test. TMTC—Too many to count, TFTC—Too few to count.
